# Supplementary material for: AffyMAPSDetector: a software tool to characterize Affymetrix GeneChip™ expression arrays with respect to SNPs
Source: BMC Bioinformatics. 2007 Jul 30;8:276. doi: 10.1186/1471-2105-8-276 (PMC1959249; doi:10.1186/1471-2105-8-276)
Supplement: Additional file 13 — AffyMAPSDetector v1 source code. AffyMAPSDetector source code. [file 1471-2105-8-276-S13.zip › AffyMAPSDetector_v1_src/doc/affychip/snpdetector/model/SnpInfo.html]

SnpInfo


|  |  |  |  |
| --- | --- | --- | --- |
| **Overview** \| **Package** \| **Class** \| **Use** \| **Tree** \| **Deprecated** \| **Index** \| **Help** | | |  |
| **PREV CLASS**   **NEXT CLASS** | **FRAMES**    **NO FRAMES**     **All Classes** |  |  |
| SUMMARY: NESTED \| FIELD \| CONSTR \| METHOD | DETAIL: FIELD \| CONSTR \| METHOD |  |  |


---


## affychip.snpdetector.model Class SnpInfo

```
java.lang.Object
  affychip.snpdetector.model.SnpInfo
```

---

public class **SnpInfo** extends java.lang.Object

Title: Probe SNP Detector

Description: This class encapsulates information about a SNP in a given
gene, e.g. GI, SNP Location, Orientation (+/-), dbSNP reference cluster ID,
Protien Accession Number, Function of the SNP (if available), SNP Class,
Alleles, and hetero-zygosity.

Copyright: Copyright (c) 2004

Company: George Mason University

**Version:**
:   1.1

**Author:**
:   Sunita Kumari

---

|  |  |
| --- | --- |
| **Field Summary** | |
| `static java.lang.String` | `delimiter` |
| `static java.lang.String[]` | `HEADER` |


**Constructor Summary**

`SnpInfo(java.lang.String accNo)`


|  |  |
| --- | --- |
| **Method Summary** | |
| `java.lang.String` | `getAlleles()` |
| `java.lang.String` | `getAxisOrientation()` |
| `java.lang.String` | `getFunction()` |
| `static java.lang.String` | `getHeader()` |
| `java.lang.String` | `getHeteroZygosity()` |
| `java.lang.String` | `getNucleotideAccNo()` |
| `java.lang.String` | `getProtAccNo()` |
| `java.lang.String` | `getRsClusterId()` |
| `java.lang.String` | `getSnpClass()` |
| `java.lang.String` | `getSnpLoc()` |
| `void` | `setAlleles(java.lang.String alleles)` |
| `void` | `setAxisOrientation(java.lang.String axisOrientation)` |
| `void` | `setFunction(java.lang.String function)` |
| `void` | `setHeteroZygosity(java.lang.String heteroZygosity)` |
| `void` | `setNucleotideAccNo(java.lang.String nucleotideAccNo)` |
| `void` | `setProtAccNo(java.lang.String protAccNo)` |
| `void` | `setRsClusterId(java.lang.String rsClusterId)` |
| `void` | `setSnpClass(java.lang.String snpClass)` |
| `void` | `setSnpLoc(java.lang.String snpLoc)` |
| `java.lang.String` | `toString()` |

**Methods inherited from class java.lang.Object**

`clone, equals, finalize, getClass, hashCode, notify, notifyAll, wait, wait, wait`

**Field Detail**

### HEADER

```
public static final java.lang.String[] HEADER
```

---


### delimiter

```
public static java.lang.String delimiter
```


**Constructor Detail**

### SnpInfo

```
public SnpInfo(java.lang.String accNo)
```


**Method Detail**

### getAxisOrientation

```
public java.lang.String getAxisOrientation()
```

---


### getFunction

```
public java.lang.String getFunction()
```

---


### getHeteroZygosity

```
public java.lang.String getHeteroZygosity()
```

---


### getNucleotideAccNo

```
public java.lang.String getNucleotideAccNo()
```

---


### getProtAccNo

```
public java.lang.String getProtAccNo()
```

---


### getRsClusterId

```
public java.lang.String getRsClusterId()
```

---


### getSnpLoc

```
public java.lang.String getSnpLoc()
```

---


### getSnpClass

```
public java.lang.String getSnpClass()
```

---


### getAlleles

```
public java.lang.String getAlleles()
```

---


### setAxisOrientation

```
public void setAxisOrientation(java.lang.String axisOrientation)
```

---


### setFunction

```
public void setFunction(java.lang.String function)
```

---


### setHeteroZygosity

```
public void setHeteroZygosity(java.lang.String heteroZygosity)
```

---


### setNucleotideAccNo

```
public void setNucleotideAccNo(java.lang.String nucleotideAccNo)
```

---


### setProtAccNo

```
public void setProtAccNo(java.lang.String protAccNo)
```

---


### setRsClusterId

```
public void setRsClusterId(java.lang.String rsClusterId)
```

---


### setSnpLoc

```
public void setSnpLoc(java.lang.String snpLoc)
```

---


### setSnpClass

```
public void setSnpClass(java.lang.String snpClass)
```

---


### setAlleles

```
public void setAlleles(java.lang.String alleles)
```

---


### toString

```
public java.lang.String toString()
```

---


### getHeader

```
public static java.lang.String getHeader()
```


---


|  |  |  |  |
| --- | --- | --- | --- |
| **Overview** \| **Package** \| **Class** \| **Use** \| **Tree** \| **Deprecated** \| **Index** \| **Help** | | |  |
| **PREV CLASS**   **NEXT CLASS** | **FRAMES**    **NO FRAMES**     **All Classes** |  |  |
| SUMMARY: NESTED \| FIELD \| CONSTR \| METHOD | DETAIL: FIELD \| CONSTR \| METHOD |  |  |


---
